# Language barriers for primary care access in Europe: a systematic review: Priyamvada Paudyal

**DOI:** 10.1093/eurpub/ckac129.724

**Published:** 2022-10-25

**Authors:** R Evenden, N Singh, S Sornalingam, S Harrington, P Paudyal

**Affiliations:** Primary Care and Public Health, Brighton and Sussex Medical School, Brighton, UK

## Abstract

**Background:**

A recent increase in migration both inside Europe and from non-European countries has expanded the language profile of many European countries. As a result, there has been a significant increase in barriers to healthcare access experienced by linguistic minority migrants. This systematic review aims to explore language barriers in primary care, focusing on the experiences of linguistic minority migrants living in Europe.

**Methods:**

PubMed, Embase, Scopus and Global Health were searched to identify studies published in English until May 2021. Qualitative and mixed methods studies on either linguistic minority migrants or healthcare workers working with linguistic minority migrants, exploring the impacts of language barriers in a primary care setting published were eligible. The studies were analysed using a Braun and Clarke's thematic analysis approach. Quality of the studies were assessed using the Mixed Methods Appraisal Tool.

**Results:**

16 studies from 14 different European countries were eligible. Participants in the studies included four groups: linguistic minority migrants (n = 11), healthcare workers (n = 10), interpreters (n = 1) and administrative staff (n = 1). Barriers identified included a lack of interpreters, limited cultural competence of practitioners, a lack of practitioner training and knowledge, a lack of accessible information for migrants, difficulties expressing emotions and building patient-practitioner relationships, and risks to women's bodily autonomy resulting from language barriers.

**Conclusions:**

Linguistic minority migrants living in Europe face a number of barriers when accessing primary care. These barriers can risk patient safety, reduce the likelihood of seeking healthcare services, and impact patient experiences of healthcare services. There is a need for improved interpreter services, practitioner training, and information accessibility for both migrants and healthcare staff.

**Key messages:**

• Linguistic minority migrants experience significant barriers to primary healthcare access across Europe.

• There is a need for improved interpreter services, practitioner training, and information accessibility for both migrants and healthcare staff.

